# Maximum entropy models of neuronal populations at and off criticality

**Published:** 2025-11-20

**Authors:** T. S. A. N. Simões, F. Lombardi, D. Plenz, H. J. Herrmann, L. de Arcangelis

**Affiliations:** 1 University of Campania “Luigi Vanvitelli”, Department of Mathematics and Physics, Caserta, Viale Lincoln, 5, 81100, Italy; 2 Department of Biomedical Sciences, University of Padova, Padova 35131, Italy; 3 Section on Critical Brain Dynamics, National Institute of Mental Health, Porter Neuroscience Research Center, Rm 3A-1000, 35 Convent Drive, Bethesda, MD, 20892, USA; 4 Universidade Federal do Ceará, Departamento de Física, Fortaleza, Ceará, 60451-970, Brazil; 5 ESPCI, PMMH, Paris, 7 quai St. Bernard, 75005, France

## Abstract

Empirical evidence of scaling behaviors in neuronal avalanches suggests that neuronal populations in the brain operate near criticality. Departure from scaling in neuronal avalanches has been used as a measure of distance to criticality and linked to brain disorders. A distinct line of evidence for brain criticality has come from thermodynamic signatures in maximum entropy (ME) models. Both of these approaches have been widely applied to the analysis of neuronal data. However, the relationship between deviations from avalanche criticality and thermodynamics of ME models of neuronal populations remains poorly understood. To address this question, we study spontaneous activity of organotypic rat cortex slice cultures in physiological and drug-induced hypo- or hyper-excitable conditions, which are classified as critical, subcritical and supercritical based on avalanche dynamics. We find that ME models inferred from critical cultures show signatures of criticality in thermodynamic quantities, e.g. specific heat. However, such signatures are also present, and equally strong, in models inferred from supercritical cultures—despite their altered dynamics and poor functional performance. On the contrary, ME models inferred from subcritical cultures do not show thermodynamic hints of criticality. Importantly, we confirm these results using an interpretable neural network model that can be tuned to and away from avalanche criticality. Our findings indicate that maximum entropy models correctly distinguish subcritical from critical/supercritical systems. However, they may not be able to discriminate between avalanche criticality and supercriticality, although they may still capture a number of important features from neuronal data.

## I. INTRODUCTION

Biological neural networks need to perform complex functions and continuously adapt. Such abilities rely on cooperative effects among local and distributed neuronal populations, which underlie the emergence of a variety of collective behaviors in the brain. The analogy between collective behaviors of populations of neurons and cooperative phenomena in physical systems undergoing a phase transition suggests that brain networks self-organize to operate at or near criticality [1], a state that provides several functional advantages [2, 3]. This hypothesis is primarily supported by observations of long-range spatio-temporal correlations and neuronal avalanches across species and spatial scales. Neuronal avalanches are cascades of local synchronized activity whose size and duration distributions follow power law behaviors, a hallmark of criticality that imply absence of characteristic scales. First identified in acute slices and longterm slice cultures of rat cortex *in vitro* [4], neuronal avalanches have since been reported *in vivo* in rats [5], monkeys [6], and other species [7, 8], including MEG and EEG recordings of the human brain [9–11]. Concomitant evidence of tuning to criticality in neuronal systems has come from maximum entropy (ME) models of neural activity, which focused on thermodynamic aspects such as the divergence of the specific heat [7, 12–15].

ME modeling has proven a powerful approach to study the dynamics of biological neural networks [16–18]. Spiking activity of neurons can be described as a binary process, σ(t), in which σ=±1 represents the state of the neuron at a given time t, i.e. spiking for σ=1 and silent for σ=−1 [19]. This approach allows one to define an empirical distribution of binary activity patterns, P(σ), which specifies the probability of observing a given spiking pattern in a population of neurons. For N binary neurons, the distribution P(σ) for all $2^{N}$ possible spiking patterns (or states) fully characterizes population dynamics. However, estimating P(σ) directly from data is often impractical since the number of possible states grows exponentially with the number of neurons [20]. Maximum entropy modeling offers a possible solution to this problem by constructing the least-biased probability distribution that matches selected statistics, such as individual firing rates and pairwise correlations, while maximizing the entropy of P(σ). The resulting distribution is mathematically equivalent to the Boltzmann distribution [16, 17, 21], which can then be analyzed using thermodynamic tools, such as the fluctuation-dissipation theorem [12, 15, 22].

This procedure can be interpreted as building a thermodynamics-like framework to describe steady-state properties (e.g., time-averaged firing rates in neural networks) of non-equilibrium systems [23, 24]. ME modeling has been extensively applied to neuronal data, such as evoked activity in the salamander retina [12, 16, 18, 25], the nervous system of the C. Elegans [26], and *in vivo* and *in vitro* neuronal activity from cortical tissue of rodents [15]. In this context, criticality is identified from a maximum in the susceptibility or in the specific heat of the data-inferred model close to unit temperature, corresponding to the observed statistics of neural activity. Such signatures of criticality in neural data can be considered static as opposed to those coming from scaling of neuronal avalanches and long-range correlations, which are intrinsically dynamic. Recently, ME models that take into account temporal dynamics of neural activity have been proposed to enhance (static) evidence of criticality, while matching avalanche statistics [13].

However, to what extent static and dynamic signatures of criticality in neural systems need to coexist and agree in baseline normal condition and how they change when such systems are perturbed away from baseline condition remains poorly understood. Are deviations from dynamical criticality captured by changes in thermodynamic properties of the corresponding maximum entropy models and vice versa? As criticality is increasingly used as a biomarker for brain disorders, these questions become of key relevance also in neuroscience.

Here, we address them by analyzing neural cultures in baseline and pharmacologically perturbed conditions, together with an interpretable neural network model whose dynamics can be tuned to criticality [22, 27]. We consider three sets of neuronal cultures: one in baseline, physiological condition, one with reduced neural excitability, and one with reduced neural inhibition [28]. Cultures in physiological condition showed neuronal avalanches whose size and duration distributions were consistent with power-laws, and were classified as critical in [28]. In cultures with reduced excitability instead, avalanches were small and short-lived, with exponential size and duration distributions. These cultures were identified as subcritical. Conversely, disinhibited cultures exhibited high excitability and a pronounced increase in the probability of very large avalanches—of the order of the system size—, leading to bimodal-like size/duration distributions. These cultures were classified as supercritical [28]. Importantly, each of these scenarios can be reproduced with our network model by tuning a single parameter [27].

We use a maximum entropy approach to model experimental and numerical data by constraining firing rates, pairwise correlations, and the distribution of synchrony, P(K), defined as the probability for K sites of the network or, alternatively, K neurons, to be active simultaneously [18]. By studying their thermodynamic properties, we show that the inferred models (mostly) agree with the dynamical classification of hypoexcitable cultures as subcritical, and correctly distinguish them from dynamically critical/supercritical cultures. However, they are not able to discriminate between dynamical criticality and super-criticality. This results are confirmed by the analysis of our integrate-and-fire (IF)neural network, whose inferred ME model closely matches the one inferred from neuronal cultures—despite the very simplified structure of the IF neural network.

This paper is organized as follows. In Sec. II, the neural network model, the experimental setup and data, and the quantities considered for the ME modeling are described. In Sec. III, the ME modeling method is explained and the inferred ME distributions are analyzed for both the simulated and experimental datasets of neuronal dynamics. Finally, Sec. IV summarizes and discusses the results.

## II. DATA ACQUISITION AND METHODS

### A. Integrate-and-fire neuronal network model

We consider an integrate-and-fire (IF) model on a scale-free, directed network with short- and long-term plasticity, and refractory period of one timestep during which neurons remain inactive after firing [27]. We implement networks with different number of neurons, N, placed within a cube of side L, but keep the density $N/L^{3}=0.016$ constant [29]. A fraction $p_{\text{in}}=20\text{\%}$ of neurons is inhibitory [30]. The out-degree k of each neuron follows a power-law distribution, $P(k)\propto k^{-2}$, with k∈[2,20] for systems with N≤100 and k∈[2,100] otherwise. The connection probability between two neurons decays exponentially with the Euclidean distance r, $P(r)\propto e^{-r/r_{0}}$, where $r_{0}=5$ [31]. The resting potential of each neuron i is set at $v_{i}=0$. A neuron fires when $v_{i}\geq v_{c}=1$, transmitting signals to all its post-synaptic neurons j according to the following equation:

(1) $$v_{j}(t+1)=v_{j}(t)\pm v_{i}(t)u_{i}(t)g_{ij},$$

(2) $$u_{i}(t+1)=u_{i}(t)\cdot (1-\delta u),$$

(3) $$v_{i}(t+1)=0,$$

where + and − are for excitatory and inhibitory pre-synaptic neurons, $g_{ij}$ is the strength of the synapsis connecting i to j, and $u_{i}$ indicates the synaptic resources of the pre-synaptic neuron i. The constant δu=0.05 controls the fraction of neurotransmitters released [32]. Here, the synaptic resources $u_{i}(t)\in [0,1]$ and the synaptic strengths $g_{ij}\in \left[10^{-5},1\right]$ change in time to model the short- and long-term plasticity, respectively.

We start our simulations with the synaptic strengths uniformly distributed in the interval $g_{ij}\in [0.4,0.6]$ and with $u_{i}=1$ for all neurons. To sustain network activity, a small external input δv=0.1 is added to a random neuron at every timestep [33]. A timestep corresponds to the time interval between the generation of the action potential at the pre-synaptic neuron and the change in the membrane potential at the postsynaptic one, and is of the order of ten milliseconds [34–36]. When a neuron reaches the threshold $v_{c}$, it fires an action potential and propagates activity to other neurons, making an avalanche start. An avalanche ends as soon as $v<v_{c}$ for all neurons. Triggering of subsequent avalanches is ensured by the small external input δv.

After each avalanche, the pool of neurotransmitters of each neuron, $u_{i}$, is recovered by an amount $\delta u_{\text{rec}}$, i.e. $u_{i}(t)\to u_{i}(t)+\delta u_{\text{rec}}$. Recovery is implemented between avalanches according to the separation of time scales in self-organized models, which considers avalanches as almost instantaneous events. For a given N, the system can be set to criticality by tuning $\delta u_{\text{rec}}$ to a certain value $\delta u_{\text{rec}}^{\text{crit}}(N)$. At criticality, the system exhibits avalanches with size S and duration D distributed according to power-laws whose cut-off scales with the system size N. Setting $\delta u_{\text{rec}}<\delta u_{\text{rec}}^{\text{crit}}(N)$ leads to subcritical dynamics, characterized by an exponential decay in the distributions of avalanche sizes, P(S), and avalanche durations, P(D). Conversely, for $\delta u_{\text{rec}}>\delta u_{\text{rec}}^{\text{crit}}(N)$ the system is in a super-critical state where there is a sharp increase in large and long avalanches, leading to the appearance of local maxima in P(D) and in P(S) around the power-law cut-off (see supplementary Fig. S1) for the distributions and Table S1 for the set of values $\delta u_{\text{rec}}^{\text{crit}}$). For simulations of subcritical and supercritical IF networks, we set $\delta u_{\text{rec}}(N)=0.1\delta u_{\text{rec}}^{\text{crit}}(N)$ and $\delta u_{\text{rec}}(N)=10\delta u_{\text{rec}}^{\text{crit}}(N)$, respectively.

#### Long-term adaptation.

The long-term plastic adaptation routine works as follow. We increase the strength of the synapses $g_{ij}$ proportionally to the voltage variation induced in the post-synaptic neuron j due to i as $g_{ij}(t+1)=g_{ij}(t)+\delta g_{j}$, where $\delta g_{j}=\beta \left|v_{j}(t+1)-v_{j}(t)\right|$, β=0.04 setting the rate of the long-term adaptation. Then, at the end of each avalanche, we decrease all $g_{ij}$ by the average increase in strength per synapse, $g_{ij}(t+1)=g_{ij}(t)-\frac{1}{N_{s}}\sum \delta g_{j}$, where $N_{s}$ is the number of synapses. Synapses that are rarely active tend to weaken over time [37]. For each IF network configuration, before performing measurements, we apply long-term plasticity rules for 10^4^ avalanches or until a $g_{ij}$ first reaches the value $g_{\min}=10^{-5}$. In this way, we shape the distribution of synaptic strengths according to the rules of Hebbian plasticity [38].

For each system size N and state of the dynamics (subcritical, critical, and supercritical), we generate five independent time series of neuronal spikes, each corresponding to a different realization of the network configuration. We refer the reader to the SI for a comprehensive account of the avalanche size and duration distributions in each network state (Fig. S1).

### B. Experimental methods and data

The data analyzed here were selected from a set of recordings for a previously published study [28], in which further details can be found. Organotypic coronal slices of rat somatosensory cortex (350 μm thick, postnatal day 0–2; Sprague Dawley), co-cultured with midbrain tissue (ventral tegmental area; 500 μm thick), were maintained on a planar 8 × 8 microelectrode array as described in [28]. Of the 64 microelectrodes on the array, the four corner electrodes were excluded from the analysis, leaving N=60 active channels for actual data collection. To induce a supercritical state, the GABA_a_ receptor antagonist picrotoxin (PTX) was added to the culture medium. To induce a subcritical state instead, two types of glutamate receptor antagonists were applied to reduce excitatory transmission. Cultures were treated either with (2R)-amino-5-phosphonovaleric acid (AP5), an NMDA receptor antagonist, or with a combination of AP5 and 6,7-dinitroquinoxaline-2,3-dione (DNQX), an AMPA receptor antagonist. Cultures without any drug treatment (baseline) were identified as being in a critical state. Using a recording head stage inside the incubator (MEA1060 w/blanking circuit; ×1200 gain; bandwidth 1–3000 Hz; 12 bit A/D; range 0–4096 mV; Multi Channel Systems), the local-field potential (LFPs; 4 kHz sampling rate; reference electrode in bath) was obtained from one hour long recordings of extracellular activity (low-pass, 100 Hz, phase-neutral) of the same culture. For each electrode, negative peaks in the LFP exceeding four standard deviations of the electrode noise were identified as firing events, and their timing was recorded. In this work, 15 recordings were analyzed, 5 for each condition: critical, subcritical (1 with AP5, 4 with mixed AP5/DNQX), and supercritical. Distributions of avalanche sizes extracted from the whole set of recordings are reproduced from [28] and shown in Fig. S2 of the SI.

### C. Time-averaged firing statistics

For the experimental datasets, each of the approximately one hour long recordings is divided into time bins of duration $\text{Δ}t_{b}=25\;\text{ms}$, within the range of values typically used in previous MEM studies on neuronal data [12, 13, 16, 18, 39], giving a total of $N_{b}\approx 1.4\cdot 10^{5}$ time bins for each recording. In the model, we monitor the firing dynamics for $N_{b}=10^{7}$ time bins, each of duration $\text{Δ}t_{b}=5$ time steps. Because the numerical timestep is on the order of a few milliseconds, the time bin for numerical data is approximately equivalent to that used for the experimental data. For each electrode on the MEA and neuron in the model, a binary variable $\sigma_{i}(k)\in \{-1,1\}$ is assigned to each bin k, with $\sigma_{i}(k)=1$ if the neuron or electrode i fires at least once in the bin k and $\sigma_{i}(k)=-1$ otherwise.

To characterize the firing dynamics, we consider three quantities: the time-averaged local activity of each neuron or electrode i, the two-point correlation function of each pair i and j, and the probability of synchrony, P(K). The time-averaged activity of each neuron or electrode i is defined as

(4) $$\langle \sigma_{i}\rangle =\frac{1}{N_{b}}\sum_{k=1}^{N_{b}}\sigma_{i}(k),$$

which is closely related to the firing rate $r_{i}=\left(\langle \sigma_{i}\rangle +1\right./2\text{Δ}t_{b}$ [18]. A related quantity is the average two-point activity correlation between all N·(N−1)/2 distinct pairs of neurons or electrodes i and j,

(5) $$\langle \sigma_{i}\sigma_{j}\rangle =\frac{1}{N_{b}}\sum_{k=1}^{N_{b}}\sigma_{i}(k)\sigma_{j}(k).$$

Together with $\langle \sigma_{i}\rangle$ (Eq. (4), these quantities define the two-point correlation functions

(6) $$C_{ij}=\langle \left(\sigma_{i}-\langle \sigma_{i}\rangle \right)\cdot \left(\sigma_{j}-\langle \sigma_{j}\rangle \right)\rangle =\langle \sigma_{i}\sigma_{j}\rangle -\langle \sigma_{i}\rangle \langle \sigma_{j}\rangle ,$$

which quantify the tendency of i and j to fire simultaneously. Finally, the probability P(K) for K∈[0,N] neurons/electrodes to fire simultaneously during the same time bin is given by

(7) $$P(K)=\frac{1}{N_{b}}\sum_{k=1}^{N_{b}}\delta_{K,K^{\prime }(k)},$$

where $\delta_{K,K^{\prime }(k)}$ is the Kronecker delta function and $K^{\prime }(k)=\sum_{i=1}^{N}\left(\sigma_{i}(k)+1\right)/2$ counts the number of neurons or electrodes that fired during time bin k. Notice that, since P(K) is a distribution, only N out of the N+1 values of P(K) are independent because of the normalization condition $\sum_{K=0}^{N}P(K)=1$.

#### 1. Allometry of firing rates

The firing rates $r_{i}=\left(\langle \sigma_{i}\rangle +1\right)/2\text{Δ}t_{b}$ define the total firing rate $\langle n_{a}\rangle =\sum_{i=1}^{N}r_{i}$, an interesting quantity that exhibits allometric scaling—a non-trivial scaling relationship—with the number of neurons, $\langle n_{a}\rangle \propto N^{\eta }$, with an allometric exponent η<1 [40, 41], which can be derived from the finite-size scaling of neuronal avalanches [40]. In the IF model, even if the network operates off criticality, $\langle n_{a}\rangle$ scales sublinearly with N (SI, Fig. S3), with a slightly lower or higher allometric exponent if the dynamics is subcritical or supercritical, respectively. The main difference between the curves is the absolute value $\langle n_{a}\rangle$, larger in the supercritical networks and smaller in subcritical ones (SI, Fig. S3). Provided that the average quiet time between successive avalanches does not diverge faster than the average avalanche duration in the limit N→∞ (SI, Fig. S4), the robustness of the allometric exponent across the states of the dynamics could be explained by the fact that avalanche size and duration distributions always have an intermediate power-law regime with similar exponents in the IF model (see supplementary Fig. S5), with a cutoff that scales similarly with N, independently of the tuning parameter $\delta u_{\text{rec}}$. However, it is important to note that in the subcritical regime the distributions are nevertheless closer to exponential-like, decaying well before reaching system-size avalanches, while in the supercritical regime the distributions show an increased probability of large, system-spanning avalanches, as evidenced by a local maximum near the system size.

#### 2. Population firing rates

**FIG. 1.**
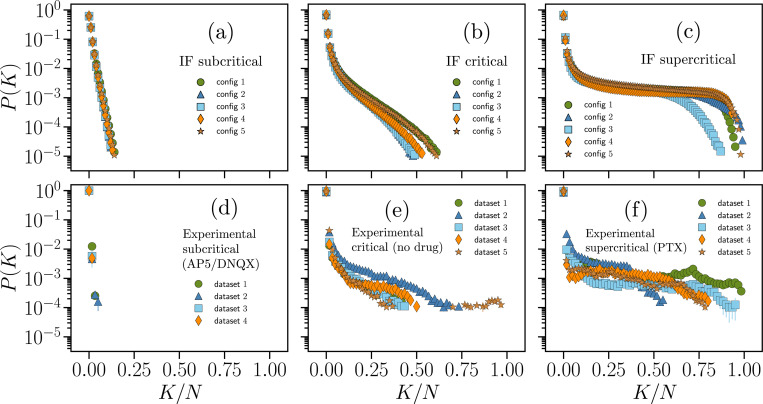
Distributions of synchrony for IF model and cortex slice cultures. Distribution P(K) (Eq. (7)) for IF networks with N=100 neurons in the subcritical (a), critical (b), and supercritical (c) state, as well as for experimental recordings of cultures treated with a combination of AP5/DNQX (d), cultures in baseline condition (e), and cultures treated with PTX (f). These cultures were respectively classified as subcritical, critical and supercritical in [28]. Different symbols correspond to either distinct network configurations of the IF model (a-c) or different neuronal cultures (d-f). Error bars represent the standard error of the mean, estimated from 100 independent subsets of the $N_{b}$ samples, and is overall smaller or equal to the symbols size.

In Fig. 1, we present the distributions of P(K), Eq. (7), for both data from the IF model and experimental recordings in the subcritical, critical, and supercritical regimes. In the IF model data, we observe that P(K) exhibits distinctive features depending on the dynamical state (Figs. 1a–c). For subcritical networks (Fig. 1a), P(K) decays rapidly, indicating low synchronous activity in the network, consistent with the exponential decay in the distributions of avalanche size and duration (Fig. S1d,i). For critical networks (Fig. 1b), the decay of P(K) is comparatively slower and non-exponential, showing that in the critical state synchronous neuronal activations (spikes) can occur involving a large fraction of the network. The distribution P(K) further broadens in the supercritical state (Fig. 1c), and shows a very slow decay over intermediate values of K/N (up to 0.75), decaying rapidly only near the system size K=N (i.e., K/N=1). This behavior reflects a pronounced increase in synchronous neural activity that can span the entire network, as also indicated by the sharp maximum at the cut-off in the distributions of avalanche sizes and durations (Figs. S1b,g).

We observe a similar behavior across the different dynamical states of neuronal cultures (Figs. 1d–f), with some more variability in critical and supercritical cultures compared to the numerical data. Notably, the probability of silence during a time bin, P(K=0), is significantly higher in the experimental data (P(K=0)∼0.86−0.99) than in the IF model (P(K=0)∼0.57−0.69). Interestingly, when considering numerical or experimental data separately, P(K=0) is similar across dynamical states.

## III. MAXIMUM ENTROPY MODELING

As introduced in IIC, the state of a neuron or electrode i is represented by the binary variable $\sigma_{i}$. Therefore, the state of the system, i.e. our neural network or culture, can be represented by a N—dimensional variable $\sigma =\left\{\sigma_{1},\sigma_{2},\ldots ,\sigma_{N}\right\}$ at each time. Let us denote by $P_{\text{data}}(\sigma )$ the probability of finding the system in one of the $2^{N}$ possible states. The structure of $P_{\text{data}}(\sigma )$ characterizes the properties of the system, but sampling all the $2^{N}$ possible states is infeasible even for moderately small networks. Alternatively, we can define $P_{\text{data}}(\sigma )$ in a way that is consistent with a given set of $N_{c}\ll 2^{N}$ time-averaged expectation values $\langle f_{m}\rangle =\sum_{\sigma }f_{m}(\sigma )P_{\text{data}}(\sigma )\approx \frac{1}{N_{b}}\sum_{k=1}^{N_{b}}f_{m}(\sigma (k))$, for large $N_{b}$, where each $f_{m}(\sigma )$ can be measured in our datasets and $\sum_{\sigma }$ indicates a sum over all possible firing states σ. This amounts to finding a probability distribution $P_{\text{MEM}}(\sigma )$ that maximizes the entropy $𝓢=-\sum_{\sigma }P_{\text{MEM}}(\sigma )\ln\left[P_{\text{MEM}}(\sigma )\right]$, subject to the constraints $\langle f_{m}\rangle =\sum_{\sigma }f_{m}(\sigma )P_{\text{MEM}}(\sigma )$ [42]. To solve this problem, we can use the method of Lagrangian multipliers [43]. For each of the $N_{c}$ constraints $\langle f_{m}\rangle$, we have an associated Lagrangian multiplier $\lambda_{m}$, and an additional one, $\lambda_{0}$, is needed to impose the normalization condition $\sum_{\sigma }P_{\text{MEM}}(\sigma )=1$. The Lagrangian then reads,

(8) $$𝓛\left[P_{\text{MEM}}(\sigma )\right]=-\sum_{\sigma }P_{\text{MEM}}(\sigma )\ln\left[P_{\text{MEM}}(\sigma )\right]+\lambda_{0}\cdot \left(\sum_{\sigma }P_{\text{MEM}}(\sigma )-1\right)+\sum_{m=1}^{N_{c}}\lambda_{m}\cdot \left(\sum_{\sigma }P_{\text{MEM}}(\sigma )f_{m}(\sigma )-\langle f_{m}\rangle \right).$$

Solving this problem involves finding the function $P_{\text{MEM}}(\sigma )$ for which the functional $𝓛\left[P_{\text{MEM}}(\sigma )\right]$ attains an extremum. The solution is mathematically identical to a generalized Boltzmann distribution with temperature T=1 (in units of the Boltzmann constant $k_{B}=1$) [12, 22]

(9) $$P_{\text{MEM}}(\sigma )=\frac{1}{Z}e^{-H(\sigma )}$$

(10) $$Z=\sum_{\sigma }e^{-H(\sigma )},$$

where one can recognize $H(\sigma )\equiv -\sum_{m=1}^{N_{c}}\lambda_{m}f_{m}(\sigma )$ as a generalized Hamiltonian and Z as the corresponding generalized partition function. The next step consists in finding the $\lambda_{m}$ that reproduce the measured expectation values from the data, which is known as the inverse Ising problem [42]. In principle, each parameter $\lambda_{m}$ can be determined from the derivative of the logarithm of the partition function (10), $\langle f_{m}\rangle =\partial \ln[Z]/\partial \lambda_{m}$. However, solving this equation exactly becomes impractical when N≳20, as the number of terms in Z grows exponentially as $2^{N}$. Alternatively, since we are trying to find the distribution $P_{\text{MEM}}(\sigma )$ that best describes the empirical one, $P_{\text{data}}(\sigma )$, we can impose that the set of parameters $\lambda_{m}$ minimize the so-called Kullback-Leibler divergence [42] between these distributions,

(11) $$D_{\text{KL}}\left(P_{\text{data}}(\sigma )\|P_{\text{MEM}}(\sigma )\right)=\sum_{\sigma }P_{\text{data}}(\sigma )\ln\left(\frac{P_{\text{data}}(\sigma )}{P_{\text{MEM}}(\sigma )}\right).$$

Performing the partial derivative of $D_{\text{KL}}$ with respect to the parameters $\lambda_{m}$ gives

(12) $$\frac{\partial D_{\text{KL}}}{\partial \lambda_{m}}={\langle f_{m}\rangle }_{\text{MEM}}-{\langle f_{m}\rangle }_{\text{data}},$$

where ${\langle f_{m}\rangle }_{\text{data}}\equiv \frac{1}{N_{b}}\sum_{k=1}^{N_{b}}f_{m}(\sigma (k))$ are the empirical averages measured from the data and ${\langle f_{m}\rangle }_{\text{MEM}}\equiv \sum_{\sigma }f_{m}(\sigma )P_{\text{MEM}}(\sigma )$ are the ones predicted by the ME distribution given by Eq. (9). Eq. (12) has two important implications. First, from the minimization condition $\partial D_{\text{KL}}/\partial \lambda_{m}=0$, the minimum of $D_{\text{KL}}$ is reached when ${\langle f_{m}\rangle }_{\text{MEM}}={\langle f_{m}\rangle }_{\text{data}}$, as intended. Second, it suggests a way to approach this minimum by updating each $\lambda_{m}$ proportionally to the corresponding difference $-\left({\langle f_{m}\rangle }_{\text{MEM}}-{\langle f_{m}\rangle }_{\text{data}}\right)$. A possible method to reach this minimum is a gradient-descent method called Boltzmann Machine (BM) learning [42, 44]. This algorithm consists in sampling the Boltzmann distribution (9) with a given set of parameters $\lambda_{m}$, and estimate the averages ${\langle f_{m}\rangle }_{\text{MEM}}$ using a suitable method such as the Metropolis algorithm [45, 46]. Then, the generated statistics, ${\langle f_{m}\rangle }_{\text{MEM}}$, are compared with those from data, ${\langle f_{m}\rangle }_{\text{data}}$, according to the following iterative scheme suggested by Eq. (12) [17],

(13) $$\lambda_{m}(n+1)=\lambda_{m}(n)-\theta (n)\cdot \left({\langle f_{m}\rangle }_{\text{MEM}}-{\langle f_{m}\rangle }_{\text{data}}\right),$$

where n is the iteration index and $\theta (n)\propto n^{-\alpha }$ is a decreasing learning rate, with the value of a α>0 that can be adjusted depending on N and the state of the dynamics of each dataset (see Table S2 in the SI).

### A. K-pairwise ME models

We are interested in finding the ME distribution $P_{\text{MEM}}(\sigma )$ that constrains the N average local activities $\langle \sigma_{i}\rangle$, the N⋅(N−1)/2 pairwise correlations $\langle \sigma_{i}\sigma_{j}\rangle$, which in turn constrain the correlation functions $C_{ij}$, and the synchronous firing probability distribution P(K). This corresponds to N Lagrange multipliers for $f_{i}(\sigma )=\sigma_{i}$, denoted by $h_{i}$, N⋅(N−1)/2 for $f_{ij}(\sigma )=\sigma_{i}\sigma_{j}$, denoted by $J_{ij}$, and N+1 for $f_{K}(\sigma )=\delta_{K,K^{\prime }(\sigma )}$, denoted by $V_{K}$. As mentioned previously, only N out of the N+1 possible values of P(K) are independent, so one only needs to fit at most N parameters $V_{K}$. Therefore, we set $V_{K=0}$ to zero. The Hamiltonian in (9) then reads,

(14) $$H(\sigma )=-\sum_{i}^{N}h_{i}\sigma_{i}-\frac{1}{2}\sum_{i,j\neq i}^{N}J_{ij}\sigma_{i}\sigma_{j}-\sum_{K=0}^{N}V_{K}\delta_{K,K^{\prime }(\sigma )},$$

where $K^{\prime }(\sigma )=\sum_{i=1}^{N}\left(\sigma_{i}+1\right)/2$ counts the number of up-spins in configuration σ, i.e. the number of firing neurons or electrodes in our case. Eq. (14) is mathematically equivalent to the Hamiltonian of a generalized Ising model, known formally as a K-pairwise model [18, 21], where $h_{i}$ is analogous to a local external field acting on spin i, $J_{ij}$ is an interaction constant between spins i and j, and $V_{K}$ is a potential that depends only on the total magnetization $M(\sigma )=2K^{\prime }(\sigma )-N$. We note that a higher value of $V_{K}$ indicates that the system favors states with K up-spins.

Each spin can model either the binary state of an IF neuron if the Hamiltonian parameters are inferred from IF model data, or the binary state of an electrode if the parameters are inferred from experimental data. The set of parameters $\left\{h_{i},J_{ij},V_{K}\right\}$ is then learned using the BM algorithm, being updated at each iteration according to Eq. (13), by applying simultaneously the following equations,

(15) $$h_{i}(n+1)=h_{i}(n)-\theta (n)\cdot \left({\langle \sigma_{i}\rangle }_{\text{MEM}}-{\langle \sigma_{i}\rangle }_{\text{data}}\right),$$

(16) $$J_{ij}(n+1)=J_{ij}(n)-\theta_{J}(n)\cdot \left({\langle \sigma_{i}\sigma_{j}\rangle }_{\text{MEM}}-{\langle \sigma_{i}\sigma_{j}\rangle }_{\text{data}}\right),$$

(17) $$V_{K}(n+1)=V_{K}(n)-\theta (n)\cdot \left(P{\left(K\right)}_{\text{MEM}}-P{\left(K\right)}_{\text{data}}\right),$$

where we set a slower learning rate $\theta_{J}(n)=\theta (n)/2$ for the interaction constants $J_{ij}$ to avoid impractical CPU times due to instabilities during the learning procedure, since their number $\left(\sim N^{2}\right)$ is much larger compared to the number of fields $h_{i}$ or potentials $V_{K}(\sim N)$. After n=5000 iterations of the BM, the learning rate of the potentials $V_{K}$ is modified to $\theta (n)\to \theta (n)/P{\left(K\right)}_{\text{data}}$ which we heuristically found to improve the learning by enabling a more efficient convergence of the values for the smallest P(K).

We start with $h_{i}(n=1)={\langle \sigma_{i}\rangle }_{\text{data}}$, $J_{ij}(n=1)=0$ and $V_{K}(n=1)=0$ and then iterate equations (15)–(17) typically until $n∼2\cdot 10^{5}$. At each iteration, quantities are averaged over $M_{c}=3\cdot 10^{5}$ spin configurations using the Metropolis algorithm. We disregard the first 150N Monte Carlo iterations in order to reduce correlations with the initial state. To avoid divergence issues due to poor sampling at small P(K), we only fit the $V_{K}$ associated with $P(K)>10^{-4}$ for experimental data or $P(K)>10^{-5}$ for the numerical data, and set all other $V_{K}=0$. We use a smaller threshold for numerical data because P(K) is better sampled thanks to the larger number of time bins that can be considered—experimental data instead have a limited ≈1h duration. At the end of the learning routine, we study the inferred Ising-like models with the set of fitted parameters $\left\{h_{i},J_{ij},V_{K}\right\}$ by averaging over an increased amount of spin configurations $M_{c}=3\cdot 10^{6}$, averaged over 100 random initial spin configurations, to reduce error bars.

### B. Inferred parameters of the K-pairwise Ising models

**FIG. 2.**
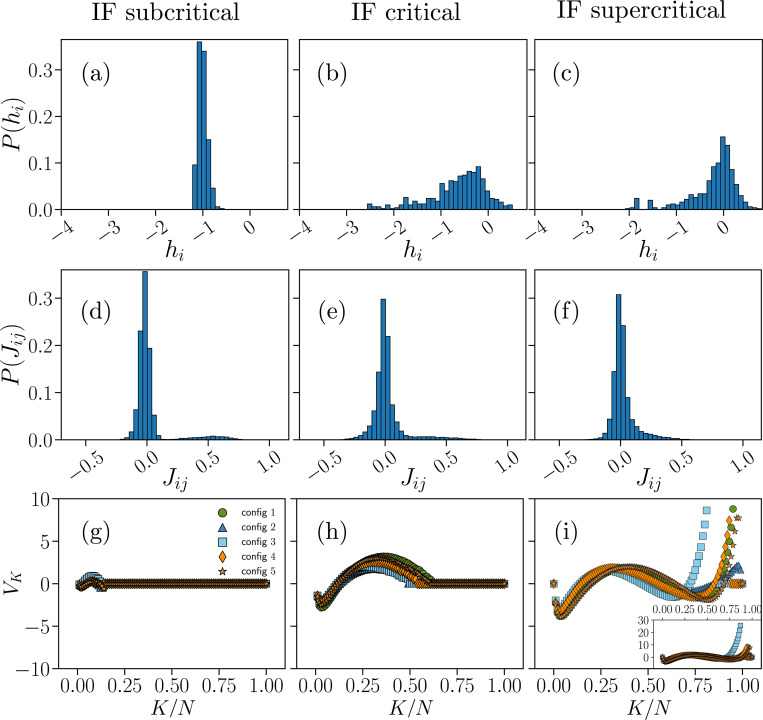
Parameters of the K-pairwise Ising-like models inferred from numerical data. Distribution of the fields $h_{i}$ (a-c), interaction constants $J_{ij}$ (d-f) and plots of the potential $V_{K}$ as a function of K (g-i), obtained from the BM learning scheme (Eqs. (15)–(17)) of the generalized Hamiltonian (Eq. (14)), for which the probability distribution $P_{\text{MEM}}(\sigma )$ from Eq. (9) has expectation values consistent with the data of the average local activities ${\langle \sigma_{i}\rangle }_{\text{data}}$, correlation functions $C_{ij}^{\text{data}}$ and synchronous probabilities $P{\left(K\right)}_{\text{data}}$ as measured in IF networks with N=100 neurons in the subcritical (left column), critical (center column) and supercritical (right column) state. The inset in (i) is a zoomed-out view of the main plot, showing the full range of $V_{K}$.

**FIG. 3.**
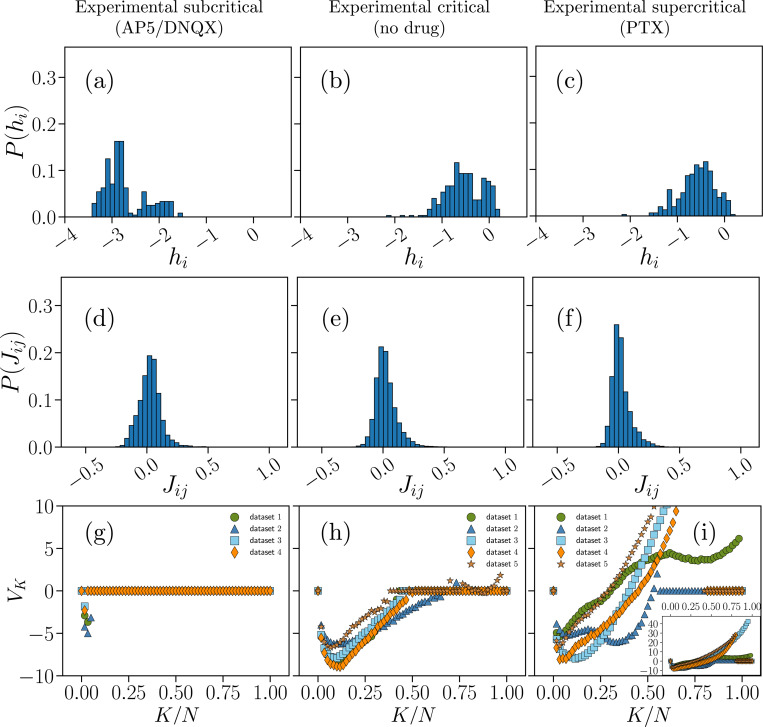
Parameters of the K-pairwise Ising-like models inferred from experimental data. Distribution of the fields $h_{i}$ (a-c), interaction constants $J_{ij}$ (d-f) and plots of the potential $V_{K}$ as a function of K (g-i) for experimental data obtained from cortex slice cultures (N=60 electrodes) treated with a combination of AP5/DNQX (left column), from baseline, no-drug cultures (center column) and cultures treated with PTX (right column), whose neuronal dynamics were respectively classified as subcritical, critical and supercritical in [28]. The inset in (i) is a zoomed-out view of the main plot showing the full range of $V_{K}$.

In Figs. 2–3 we compare the distributions of the fields $h_{i}$ (a-c), interaction constants $J_{ij}$ (d-f), and the potential $V_{K}$ as a function of K (g-i), obtained from the BM learning process on the IF model and experimental data. These parameters reproduce the original input data from IF model networks with N=100 neurons (Fig. 2) or from the N=60 electrodes of the neuronal cultures (Fig. 3) in the subcritical (left column), critical (center column), and supercritical states (right column). For the quality of the fit, we refer the reader to the SI, Figs. S6-S11.

For the model, the fields $h_{i}$ (Fig. 2a–c) are overall negative for the subcritical and the critical state with an increasing probability for positive values when moving towards the supercritical state. In the subcritical state, the $h_{i}$ are in a very narrow range around −1, which implies that neurons fire rather sparsely (Fig. S3). In critical state instead, the distribution of $h_{i}$ broadens and shifts towards zero, with non-zero probabilities for small positive $h_{i}$ (Fig. 2b), showing an increasing heterogeneity in the local fields. This indicates that, at criticality, neuronal firing is still sparse, but the network tends to be more active. Indeed, the average firing rate increases of about ten times as compared to the subcritical state (Fig. S3). When moving towards the supercritical state, the probability for positive $h_{i}$ increases significantly, signaling a consistent increase in firing activity with respect to the baseline critical state, the average firing rate being about ten times larger than in the critical state.

The results for our IF model closely recapitulate the distributions $P\left(h_{i}\right)$ learned from the experimental data (Fig. 3). The hypoexcitable cultures (AP5/DNQX), which were classified as subcritical on the basis of avalanche metrics [28], consistently show negative $h_{i}$ only, distributed between −2 and −3 (Fig. 3a). This scenario is consistent with strongly reduced firing rate in these cultures [28] (Fig. S10). In cultures treated with AP5 only (see SI, Fig. S12) the $h_{i}$ tend to be less negative and mostly close to −1. We note that AP5 acts as an NMDA receptor antagonist, suppressing the slower, more sustained form of excitatory transmission between neurons. DNQX, on the other hand, blocks AMPA receptors, which mediate fast excitatory signaling. Although AP5-treated cultures retain many characteristics of critical dynamics, only the combined inhibition of NMDA and AMPA receptors using AP5 and DNQX effectively shifts the cultures into a subcritical state by reducing their overall excitability. It is important to notice that, unlike model simulations, in this case neural dynamics tend to show more variability across samples (i.e. cultures), also in terms of distance from criticality [28]. In the baseline critical cultures, we find that $P\left(h_{i}\right)$ is remarkably close to the one inferred from numerical data, covering a range of value between −1 and 0 that is consistent with the observed increase in firing activity [28] (Fig. S9). We observe a similar distribution of $h_{i}$ in the disinhibited cultures treated with PTX and classified as supercritical [28], which however present an increased firing rate as compared to the critical case (Fig. S11).

The second set of parameters inferred from numerical and experimental data provides the interaction constants $J_{ij}$ of the Hamiltonian (Eq. 14). In both numerical (Figs. 2d–f) and experimental data (Figs. 3d–f), the distribution of $J_{ij}$ is centered around $J_{ij}\approx 0$ for all network states. We observe little or no modulations in $P\left(J_{ij}\right)$ across network states, in particular between the critical and supercritical state, both in experimental and numerical data. Notably, subcritical IF networks display a small subset of positive interaction constants (Fig. (2d)) around $J_{ij}\approx 0.5$. This mild bimodal behavior is further attenuated in the critical state, and is not present in the supercritical case, where the distribution becomes unimodal with a heavy positive tail as for experimental data.

Finally, we examine the inferred potentials $V_{K}$, which are related to the distribution of synchrony, P(K). Here, we first discuss the results for our IF model, and then compare them with the experimental data. In the subcritical state (Fig. 2g), the $V_{k}$ inferred from the model are approximately zero for all K, indicating that synchronous firing of even a small fraction of neurons is not likely in this state—as demonstrated by the distribution P(K) (Fig. 1a) and in line with evidence that collective bursts are rare in weakly excitable networks [28]. This may make global observables such as the synchronous probability P(K) less relevant in this regime.

Both the critical and supercritical states present a markedly different scenario. At criticality (Fig. 3h), the $V_{k}$ have a negative minimum at low values of K/N=0.03±0.01, where the uncertainty is estimated as the range between the minimum and maximum values obtained from the different datasets, and are positive over a range of intermediate K—with a maximum at K/N=0.35±0.10)—, which would favor synchronous co-activation of neurons consistent with transient collective bursts of activity. In the critical state indeed, the P(K) is non-zero over a broad range of K (Fig. 1b). Such features of the potentials $V_{k}$ persist in the supercritical states (Fig. 3i), and become slightly more pronounced, in particular the minimum at K/N=0.03±0.01. At the same time, the supercritical state shows a key distinctive feature in most numerical samples, namely very high values of $V_{k}$ for K/N>0.75. Consistently, the P(K) has a much longer tail (Fig. 1c). This is closely related to the sharp increase in the probability of very large avalanches in the supercritical regime (Fig. S1), and is consistent with strong co-activation of neuronal population in disinhibited networks [28, 47].

Comparison with $V_{K}$ inferred from cultures (Fig. 3g–i) shows some common features and some important differences, particularly in the critical state. In cultures treated with AP5 and DNQX $V_{K}$ is always zero, except for a localized negative value at K/N=0.02±0.01. This is in line with inference from our network model and reflects absence of collective firing in hypoexcitable cultures [28]. Accordingly, the distributions P(K) show a sharp exponential decay (Fig. 1d). All these cultures were originally classified as subcritical and showed no power-law behavior in avalanche size and duration distributions [28]. An important exception is represented by the one culture that was treated only with AP5 (SI, Fig. S12). Although classified as subcritical, the $V_{K}$ are strongly negative over a wider range of K (Fig. 3g), and P(K) exhibits a broad tail (Fig. S12)c.

This scenario closely resembles the behavior of $V_{K}$ at criticality, where negative non-zero values characterize $V_{K}$ for K/N<0.5, with a minimum K/N=0.10±0.03, and $V_{K}$ is always zero for K/N>0.5 (Fig. 3h). This confirms that only the combined inhibition of NMDA and AMPA receptors using AP5 and DNQX effectively produce a subcritical network state. We notice that, unlike in our model, $V_{K}$ is almost never positive in cultures at criticality. Yet, the distribution of synchrony, P(K), for critical cultures qualitatively matches our model at criticality (Figs. 1b, e). Moving to the supercritical state (PTX), the $V_{K}$ remain mostly negative for K/N<0.5 in all cultures (local minimum K=0.15±0.15). On the contrary, most cultures show increasing positive $V_{K}$ for K/N>0.5 (Fig. 3i). Importantly, we observed a similar behavior in our network model (Fig. 3i). As in the model, supercritical cultures are characterized by higher firing rates, a very broad distribution of neural synchrony (Fig. 1f), and an excess of large avalanches [28].

Overall, these results show that our neural network model is well described by a generalized Ising-like model that closely resembles the Ising-like model inferred from neuronal data classified as either subcritical, critical or supercritical by means of avalanche-based metrics. Importantly, our model also captures the main features of avalanche size and duration distributions at and away from criticality (Fig. S1).

### C. Predictive capability of the K-pairwise Ising models

The predictive capability of the generalized Ising models can be tested by comparing quantities not constrained by the ME modeling scheme, such as the three-point correlation functions $T_{ijk}$,

(18) $$T_{ijk}=\langle \left(\sigma_{i}-\langle \sigma_{i}\rangle \right)\cdot \left(\sigma_{j}-\langle \sigma_{j}\rangle \right)\cdot \left(\sigma_{k}-\langle \sigma_{k}\rangle \right)\rangle =\langle \sigma_{i}\sigma_{j}\sigma_{k}\rangle -\langle \sigma_{i}\rangle \langle \sigma_{j}\sigma_{k}\rangle -\langle \sigma_{j}\rangle \langle \sigma_{i}\sigma_{k}\rangle -\langle \sigma_{k}\rangle \langle \sigma_{i}\sigma_{j}\rangle +2\langle \sigma_{i}\rangle \langle \sigma_{j}\rangle \langle \sigma_{k}\rangle .$$

**FIG. 4.**
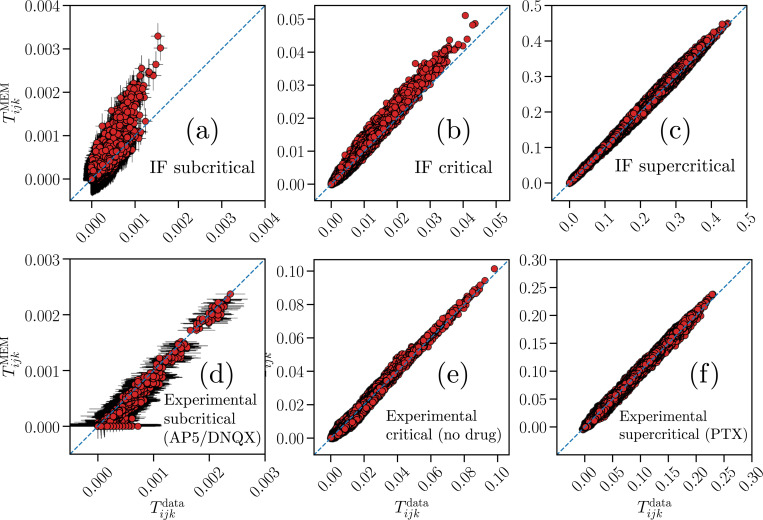
Predictive capability of the K-pairwise Ising models inferred from numerical and experimental data. Comparison of the three-point correlation functions $T_{ijk}$ between the data (x-axes) and the prediction of the maximum entropy distribution (Eq. (9)) (y-axes), for IF networks with N=100 neurons in the subcritical (a), critical (b), and supercritical (c) states, as well as experimental recordings from cultures treated with a combination of AP5/DNQX (d), cultures in baseline, no-drug condition (e) and cultures treated with PTX (f). The blue dashed lines indicate the bisector y=x. For the original data (x-axes), results are averaged over $N_{b}=10^{7}$ time bins for the IF model, and over $N_{b}\approx 1.4\cdot 10^{5}$ time bins for the experimental datasets. For the sampling of the distribution $P_{\text{MEM}}(\sigma )$ from Eq. (9) (y-axes), using the respective learned parameters shown in Fig. 2, results are averaged over $M_{c}=3\cdot 10^{6}$ spin configurations, and over 100 random initial spin configurations. Error bars represent the standard error of the mean, and, with the exception of the subcritical cases (a) and (d), are smaller than or equal to the symbol size.

In Fig. 4 the three-point correlation functions predicted by the ME distribution, $T_{ijk}^{\text{MEM}}$ (y-axes), are compared with those from the original data, $T_{ijk}^{\text{data}}$ (x-axes), for both IF model networks and neuronal cultures, in the subcritical, critical, and supercritical states. Notably, the generalized Ising models reproduce the three-point correlation functions $T_{ijk}$ most accurately for networks with stronger $T_{ijk}$, which is the case for the critical and supercritical datasets, both in the IF model (Fig. 4b–c) and in the experimental data (Fig. 4e–f). Three-point correlations are extremely weak in the subcritical state. Both in the network model and in cultures treated with AP5/DNQX, we find $T_{ijk}<10^{-3}$—more than one order of magnitude smaller than in critical and supercritical states (Fig. 4a and Fig. 4d). In constrast, for the reasons discussed above, the culture treated with AP5 shows three-point correlations similar to cultures at criticality (Fig. S12g), despite the fact that avalanche analysis suggests that it is in a subcritical state [28].

Overall, we find that the Ising-like models predict $T_{ijk}$ more accurately for the experimental data than for the neural network model. In particular, the inferred Ising-like models tend to substantially overestimate $T_{ijk}$ for the network model in the subcritical state (Fig. 4a). This may be due to the extremely small values of the $T_{ijk}$ combined with larger errors in the fitted features that are used to obtain the estimates of $T_{ijk}$. Indeed, propagation of small errors on the fitted features may lead to the observed mismatches, which are < 10^−3^.

We note that, as expected, the predictive capability improves for critical IF networks when the generalized Ising-like models include constraints on the synchronous probability P(K) compared to models constraining only the average local activities and correlation functions (compare Fig. 4b with Fig. 6 in [22]).

### D. Thermodynamics of the K-pairwise Ising models

The ME distribution (9) can be generalized by introducing an additional parameter T [12],

(19) $$P(\sigma ,T)\equiv \frac{1}{Z(T)}e^{-H(\sigma )/T},$$

with $Z(T)=\sum_{\sigma }e^{-H(\sigma )/T}$. Here, T is a temperature-like parameter in units of the Boltzmann constant $\left.\left(k_{B}=\right.1\right)$, which sets the strength of the Ising parameters $\left\{h_{i},J_{ij},V_{K}\right\}$ by uniformly rescaling them by the same factor 1/T. This parameter is useful for probing thermodynamic properties of the models, and for investigating whether they exhibit notable behaviors near T=1, where $P(\sigma ,1)=P_{\text{MEM}}(\sigma )$.

Given a set of N spins, the total magnetization $M(\sigma )=\sum_{i=1}^{N}\sigma_{i}$ and the energy H(σ) defined in Eq. (14) can be obtained for different temperatures. From the fluctuations of these quantities, according to the fluctuation-dissipation theorem, the isothermal magnetic susceptibility χ and heat capacity $C_{v}$ can be calculated as functions of T,

(20) $$\chi =\frac{1}{T}\cdot \left(\langle M^{2}\rangle -{\langle M\rangle }^{2}\right),$$

(21) $$C_{v}=\frac{1}{T^{2}}\cdot \left(\langle H^{2}\rangle -{\langle H\rangle }^{2}\right),$$

where 〈⋯〉 indicates an average over spin configurations generated by sampling Eq. (19) using Monte Carlo simulations.

**FIG. 5.**
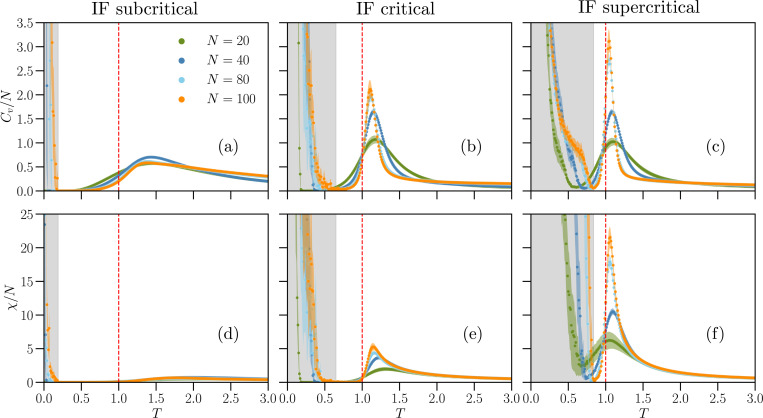
Thermodynamic response functions of the K-pairwise Ising-like models inferred from numerical data. Specific heat $C_{v}/N$ (a-c) and intensive susceptibility χ/N (d-f) as functions of temperature T∈[0.01,3.00] for K-pairwise Ising models fitted to IF networks of size N∈{20,40,80,100} in subcritical (left column), critical (center column), and supercritical states (right column). Vertical dashed lines indicate T=1, where the probability (Eq. (19)) matches the maximum entropy distribution (Eq. (9)) that fits the data. For each T, results are averaged over $M_{c}=3\cdot 10^{6}$ spin configurations, and 100 random initial spin configurations. The gray background indicates the temperature regime where Metropolis Monte Carlo results for N=100 depend on the initial spin configuration. The colored shaded areas around the curves of $C_{v}$ and χ represent the standard error obtained from 5 different IF network configurations for each N.

**FIG. 6.**
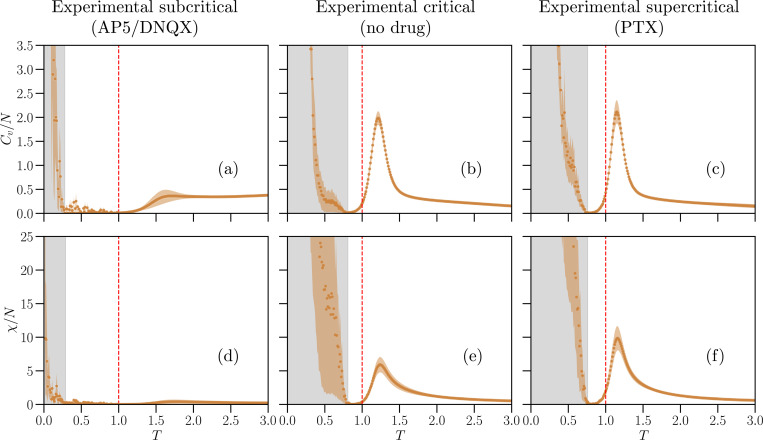
Thermodynamic response functions of the K-pairwise Ising-like models inferred from experimental data. Same as in Fig. 5 but for data recorded in cortex slice cultures (N=60 electrodes) treated with a combination of AP5/DNQX (left column), in baseline, no-drug cultures (center column) and in cultures treated with PTX (right column) The gray background indicates the temperature regime where Metropolis Monte Carlo results depend on the initial spin configuration. The colored shaded areas around the curves of $C_{v}$ and χ is the standard error obtained from 5 experimental subsamples of the same cortical culture.

In Figs. 5 and 6, we show the Monte Carlo results for the intensive susceptibility χ/N and specific heat $C_{v}/N$ as functions of the temperature T in the K-pairwise Ising models inferred from the IF neural network and from cultures at and off criticality. For the Ising-like model inferred from the IF networks, we consider several system sizes, N∈[20,100]. In cultures, we have N=60, which is the number of recording electrodes.

The K-pairwise Ising models inferred from real and IF networks in the critical state present maxima of the heat capacity and susceptibility at $T=T_{\max}$ slightly larger than one (see SI, Table S3 for the values of $T_{\max}$). In the models inferred from critical IF networks, these maxima increase faster-than-linearly with the system size N for the critical case (Figs. 5b,e), scaling as $\max\left[C_{v}\right]\propto N^{a}$ with a=1.41±0.06 and $\max[\chi ]\propto N^{b}$ with b=1.57±0.09, where the exponents were estimated from a least-square fit of the data and their errors calculated by bootstrapping. Importantly, for the critical IF model, $T_{\max}$ consistently approaches T=1 as N increases, suggesting that in the thermodynamic limit N→∞ the inferred Ising models operate at a critical point. This is consistent with the original criticality of the IF model data, and in line with results from pairwise Ising models that were inferred by constraining only $\left\{{\langle \sigma_{i}\rangle }_{\text{data}}\right\}$ and $\left\{C_{ij}^{\text{data}}\right\}$ [22].

Notably, the models inferred from supercritical IF (Fig. 5c,f) and real neuronal networks (Fig. 6c,f) also display maxima in $C_{v}$ and χ close to T=1. In this case, the maximum of $C_{v}$ increases with the number of IF neurons, N, faster than in the critical case, i.e. $\max\left[C_{v}\right]\propto N^{a}$ with a=1.70±0.06. Unlike $C_{v}$, the maximum of χ follows a scaling consistent with the critical case, i.e. $\max[\chi ]\propto N^{b}$ with b=1.77±0.12.

In contrast to critical and supercritical inferred models, subcritical inferred models show very attenuated (or non clear) maxima. Moreover, these maxima do not increase with the system size in the case of IF network models (Figs. 5a,d). We notice that $C_{v}$ and χ show very different behaviors in AP5/DNQX-treated and AP5-treated cultures (compare Fig. 6a,d to Fig. S12). While in AP5/DNQX-treated cultures the maxima in $C_{v}$ and χ are barely identifiable, similarly to the numerical data, in AP5-treated cultures $C_{v}$ and χ exhibit maxima in line with those of the critical state. This confirms that combined inhibition of NMDA and AMPA receptors using AP5 and DNQX is necessary to drive the cultures into a subcritical state.

To conclude the analysis of the thermodynamics of the inferred Ising models, we examine the low temperature behavior of $C_{v}$ and χ. For $T<T^{*}<1$, the numerical values of $C_{v}$ and χ depend on the initial spin configuration (see SI, Figs. S13 and S14). This interval $T<T^{*}$ is indicated by a gray-shaded background in Figs. 5 and 6 for the largest system sizes considered (N=100 neurons in the IF model and N=60 electrodes for experimental data). This behavior suggests the presence of a complex phase below the phase transition $T<T_{\max}$, analogous to the spin-glass phase in the Sherrington-Kirkpatrick model [48], where many frustrated spin configurations are present and thermal fluctuations are insufficient to drive the system from a random initial state to the ground state within feasible Monte Carlo simulation times [49, 50]. The divergence of $C_{v}$ and χ as T→0 is therefore a numerical artifact, likely the result of averaging over different metastable states with varying energies H(σ) and magnetizations M(σ), resulting in large values for the fluctuations in Eqs. (20) and (21). The temperature $T^{*}$ depends on the system size, increasing with N, and on the state of the dynamics of the IF networks, being smallest for subcritical IF networks and largest for supercritical ones (see SI, Fig. S13 and Table S4). For low temperature T→0, Ising-like models with disorder in the parameters, such as the Sherrington-Kirkpatrick spin glass model, exhibit metastable states separated by free-energy barriers that scale with N [51]. If K-pairwise models fitted to neuronal data would also exhibit similar free-energy barriers that scale with N at low temperature, then the shift of $T^{*}$ to higher temperatures with system size could be explained by the increasing thermal energy needed to overcome these barriers as N increases.

## IV. DISCUSSION

In this study we presented a thorough analysis of ME models inferred from cultures of neurons at and away from criticality, and compared them with equivalent models inferred from integrate-and-fire neural networks that can be tuned to operate at and away from criticality. We assumed an operational definition of criticality based on neuronal avalanche metrics, as originally defined for cultures of neurons [28]. The IF model correctly reproduces these metrics at criticality, i.e. exponents of power-law size and duration distributions, shows absence of scaling in the subcritical state (exponential-like distributions), and a sharp increase in large avalanches (order of the system size) in the supercritical state. Here, we showed that, despite the intrinsic difference in the underlying dynamics, ME models inferred from this IF neural network closely match those that are inferred from neuronal data, in particular the neuronal coupling structure and the local fields $h_{i}$ (see distributions of $J_{ij}$ and $h_{i}$ in Figs. 2 and 3).

Importantly, this close equivalence extends to thermodynamic quantities such as the specific heat, $C_{v}$, and the susceptibility χ. In Ising models inferred both from simulation and experimental data at criticality, $C_{v}$ and χ show pronounced maxima near the effective temperature T=1—the temperature at which ME models were inferred. As expected at criticality, this maxima increased with the system size. However, our results showed that such maxima persists in systems (numerical and experimental) that are classified as supercritical on the basis of avalanche metrics. On the contrary, ME models inferred from subcritical systems, both numerical and experimental, do not show such evidence of criticality. This indicates that ME models that only incorporate time-averaged quantities, i.e. no dynamics, correctly distinguish between systems classified as subcritical and critical/supercritical according to neuronal avalanche metrics. However, they may not be able to discriminate between avalanche criticality and supercriticality, although they may still capture a number of important features as we shall discuss in turn.

### A. Modulation of ME modeling parameters across network states

The local fields $h_{i}$ are key parameters in the inferred Ising models that control the excitability of neurons. We observed that the distribution of $h_{i}$ shifts from strongly negative values toward less negative and positive values as systems are driven from the subcritical to the critical and supercritical state. This trend reflects, to some extent, the increase in average firing rate observed when moving from subcritical to supercritical states, both in our network model and in neural data [28]. In neural data, a subcritical state is induced by reducing network excitability, whereas supercritical states are obtained by reducing inhibition. Both interventions alter the excitation/inhibition balance of the network, which not only affects neural firing rates, but also has a strong impact on collective, synchronous firing. This is demonstrated by the broadening of the distribution of synchrony P(K) when moving towards the critical and supercritical states (Fig. 1), and also reflected in avalanche size and duration distributions [28, 52].

A similar behavior is found in our neural network model. In the K-pairwise ME modeling, one constrains the distribution P(K), which provides, at most, an additional N potentials, $V_{K}$. When there is little or no synchronous firing across the network, as in the subcritical state, $V_{K}$ are mostly zero. On the contrary, $V_{K}$ are not negligible in critical and supercritical states. Notably, we found that $V_{K}$ are strongly altered in the transition from criticality to supercriticality, in particular at large K, where they become strongly positive—in the same range $V_{K}$ are zero at criticality. Among all the inferred parameters, the fields $V_{K}$, show the most striking difference when comparing critical and supercritical states. Because they are related to a collective variable, the modulations in $V_{K}$ can be easily translated into changes in avalanche dynamics, as outlined above.

We observed similar modulations of $V_{K}$ across network states for the neural network model and the experimental data. However, we note an important difference between the two. While $V_{K}$ are always negative or zero in data-inferred Hamiltonians and only become positive at large K in the supercritical state, in model-inferred Hamiltonians they are mostly positive or zero. This difference may arise from the model dynamics controlling the emergence of synchronous firing, which are likely to differ from those that underlie neural activity in cultures.

We note that including the constraint on the synchronous firing probability improves the prediction capability of the Ising-like model for higher-order correlations, as also reported in [18, 53]. Indeed, in [22], the P(K) was not constrained and the inferred model systematically overestimated higher-order correlations at criticality (compare Fig. 4b with Fig. 6 in [22]).

### B. Thermodynamic quantities across network states

We have shown that K-pairwise Ising models inferred from critical and supercritical systems show pronounced maxima in specific heat $C_{v}$ and susceptibility χ near the effective temperature T=1. This feature is common both to the network model and the neural cultures (Figs. 5b–c, f–g). Moreover, in our neural network model, we also demonstrated that these maxima grow superlinearly with the number of neurons in both critical and supercritical states. Overall, this evidence would suggest that, in both cases, the system is at or close to criticality. However, this would contradict their classification based on avalanche dynamics. A similar contradiction emerges when we consider individual culture treated with AP5 only, which acts as an NMDA receptor antagonist and suppresses slower and more sustained form of neuronal excitation. For this culture, both $C_{v}$ and χ showed pronounced maxima near T=1, suggesting instead a critical state (Fig. 6a, d), and indicating that only combined inhibition of NMDA and AMPA receptors drives the cultures into a subcritical state by reducing their overall excitability.

Criticality in neuronal systems has been hypothesized to optimize stimulus response and maximize function such as dynamic range, the range of stimuli that can be processed by the network to produce a functional response, information storage and capacity [3]. In [28], it was shown that cultures at criticality have a much higher dynamic range compared to sub- and supercritical cultures. The dynamic range is related to the responsiveness of the network to external stimuli, i.e. to its susceptibility. Thus, we would expect the susceptibility to be high for Ising models inferred from critical cultures and low for models inferred from sub- and supercritical cultures. Although this prediction is met for cultures treated with AP5/DNQX, classified as subcritical (except for the one treated with AP5 only), we observe a pronounced maximum in the susceptibility for supercritical cultures.

### C. Identifying criticality in neural data

Altogether, our analysis points to important differences between dynamical criticality (presence of power-law scalings in neuronal avalanches) and static criticality (maxima in $C_{v}$ and χ near the unit temperature), particularly when trying to assess deviations of neuronal cultures from the critical state.

It has been suggested that inference of Ising-like models from data using ME modeling tends to return parameters that set the inferred models close to critical points, independently of the criticality signatures that may exist in the data [54]. Previous work [55], where K-pairwise models were inferred from numerical data generated from a phenomenological model of retinal ganglion cells, indicated that the larger the firing rates and correlation functions, the more likely it is to find signatures of criticality, such as diverging specific heat, irrespective of the underlying state of dynamics in the original data. This is consistent with our results. In our neural network model, average local activities and pairwise correlations increase as the IF networks are shifted from subcritical to critical, and from critical to supercritical (SI; compare the overall values of Figs. S6a-h, S7a-h and S8a-h). Similarly, the average firing rate and pairwise correlations increase from subcritical to critical and supercritical cultures [28, 47]. Therefore, caution must be taken when drawing conclusions about criticality or deviations from criticality based on ME modeling approaches that consider only time-averaged information. ME approaches that incorporate dynamic information, such as the joint distribution of the number of spiking neurons at different time windows [13], may be more suitable for this purpose, as suggested by recent analysis of neuronal network models [56].

At low temperatures $T<T^{*}<1$ (Figs. 5 and 6), Monte Carlo results for the specific heat and the susceptibility depend on the initial spin configuration, suggesting the presence of a spin-glass-like phase characterized by the presence of many metastable states with frustrated spins. This temperature $T^{*}$ increases (i.e. move towards the unit temperature) with system size and as IF networks shift from subcritical to critical and supercritical states, and similarly for experimental data shifting from subcritical to critical/supercritical, while it remains more or less constant between the experimental critical and supercritical cases (see Fig. S13 and Table S4) in the SI). Notably, pairwise Ising models inferred from spiking data recorded from the visual, auditory, motor, and somatosensory cortices of freely moving rats, were also found to operate near the boundary of a spin-glass-like phase [50].
